# Editorial: Dynamic relationship between secretome of adipose tissue and nutrition

**DOI:** 10.3389/fendo.2023.1166914

**Published:** 2023-03-31

**Authors:** Pamela Senesi, Anna Ferrulli, Ileana Terruzzi

**Affiliations:** ^1^ Department of Biomedical Sciences for Health, Università degli Studi di Milano, Milan, Italy; ^2^ Department of Endocrinology, Nutrition and Metabolic Diseases, IRCCS MultiMedica, Milan, Italy

**Keywords:** inflammation, oxidative stress, obesity, cardiovascular disease, diet, nutrigenomics

Over past decades, nutritional and lifestyle habits, characterized by unbalanced ratio between high caloric intake and low caloric expenditure, have provided fertile ground for increasing prevalence of obesity that currently is one of the most important healthy and economic global problem (Rajamoorthi et al.).

Huge data have demonstrated that dysregulated production of adipose secretome, triggered by unbalanced adipose tissue mass, leads to the development of non-communicable diseases, including diabetes, cardiovascular and respiratory disease, cognitive decline, and cancer. Since the discovery of leptin, the first adipokine identified in 1994, characterization of adipose secretome and its molecular mechanisms represents one of the most promising therapeutic weapons in the treatment of obesity and its correlated pathologies (1).

In this Research Topic, Song et al. have observed not only that TNF-α secretion by adipocytes is correlated with ASK1-interacting protein 1 (AIP1), a signalling scaffold protein involved in inflammatory process, but also that in omental adipose biopsies of obese-diabetic subjects compared to obese patients AIP1 was downregulated. Also, Zaidi et al. have investigated the relationship between TNF-α and adiponectin expression in different fat depots, i.e. superficial, deep and visceral adipose tissue.

Image studies, gene expression analysis and proteomics have contributed and are contributing to characterize adipose tissue as an intricate endocrine organ whose secretome is determined to adipocytes features, environment inputs (i.e. diet and exercise) and pathological state. The use of term “batokines”, to distinguish bioactive molecules preferentially secreted to brown adipose tissue from adipokines produced to white adipose tissue, is only scratching the surface of this complexity. The analysis of secretome of epicardial adipose tissue (EAT) is a typical example of the extreme plasticity and functional complexity of adipose tissue.

EAT, distinctive fat depot located between the myocardium and the epicardium, secretes different cardioprotective adipokines, such as adiponectin and adrenomedullin. However numerous basic and clinical studies have demonstrated that EAT endocrinological function is related to age and cardiac health: in the early years of life EAT has brown fat-like activity, that gradually decreases with ageing or in pathological conditions, above all in end-stage of heart failure or diabetes, that enhance the secretion of pro-inflammatory, profibrotic and pro-apoptotic factors (2). But the hypothesis that EAT acts as a friend in childhood and an enemy in old age or in chronic pathological conditions is inappropriate. Indeed, recent data indicate that pharmacological modulation using novel anti-diabetic drugs, glucagon-like peptide 1 receptor (GLP1R) agonists and sodium–glucose cotransporter 2 (SGLT2) inhibitors, could re-established EAT cardioprotective role (2).

It is important to note that genomics and proteomics studies usually increase the intricate adipose secretome identifying new adipokines. In this Research Topic, Vergani et al. have demonstrated that circulating levels of neudesin, novel bioactive molecule secreted by brain and adipose tissue, were significantly higher in obese/overweight children than in controls and directly correlated with blood glucose. Similarly, Wang et al. have investigated the synergic role of the novel hepatokine, called angiopoietin-like protein 8, and leptin in the regulation of cardiac remodelling process in a cohort of young patients having a risk to development metabolic syndrome.

These two articles highlight three crucial aspects of the research focused on adipose secretome: multifaceted physiological role of adipokines, strictly interrelation with other secreted peptides and childhood and adolescent obesity.

First, numerous adipokines, as neudesin, not are only secreted by adipose tissue, but also by other tissues and consequently their action influencing not only metabolic state but also other different pathologies and indeed Novais A et al. have demonstrated that neudesin have neurotrophic action in mice adult brain (3). At the same manner, irisin, the most known adipomyokine, acts as a crucial player in adipose-muscle processes involved in age-related disorders (4).

Different adipokines mutual operate with peptides secreted from cells residing in liver or in different organs. For example, leptin and adiponectin interact with different myokines or hepatokines (5, Ren et al.) and moreover, as also reported in this Research Topic by Chen et al., regulate progesterone production.

Finally, articles of Vergani et al. and Wang et al. underline the new challenge of research: to identify and counteract the specific pathological mechanisms implicated in childhood and adolescent obesity. As known, childhood obesity is continuously increasing and induces metabolic syndrome onset and psychological pathologies, from body image dissatisfaction to depression, that in many subjects emphasize eating disorder. Moreover, childhood obesity is correlated with precocious puberty and polycystic ovary syndrome in girls, and recent data suggest that also in obese boys pubertal development can be accelerated (6).

As reported in this Research Topic by Chen et al. an altered network of adipokines characterized polycystic ovary syndrome and influences all the different phases of menstrual cycle.

In conclusion, this Research Topic highlights the evolution of “adipose secretome” concept: thirty years ago, adipose secretome was a hypothesis, currently it is a fascinate and intricate and partly still mysterious network involved in several pathophysiological conditions, in future adipose secretome will be a fundamental therapeutical target in the treatment of non-communicable diseases.

## Author contributions

PS wrote the first draft of the present manuscript. AF and IT contributed to its critical revision. All authors contributed to the article and approved the submitted version.

## References

[1] Zorena K Jachimowicz-Duda O Ślęzak D Robakowska M Mrugacz M. Adipokines and obesity. potential link to metabolic disorders and chronic complications. Int J Mol Sci (2020) 21(10):3570. doi: 10.3390/ijms21103570 32443588 PMC7278967

[2] Iacobellis G. Epicardial adipose tissue in contemporary cardiology. Nat Rev Cardiol (2022) 19(9):593–606. doi: 10.1038/s41569-022-00679-9 35296869 PMC8926097

[3] Novais A Silva A Ferreira AC Falcão AM Sousa N Palha JA. Adult hippocampal neurogenesis modulation by the membrane-associated progesterone receptor family member neudesin. Front Cell Neurosci (2018) 12:463. doi: 10.3389/fncel.2018.00463 30534059 PMC6275434

[4] Bao JF She QY Hu PP Jia N Li A. Irisin, a fascinating field in our times. Trends Endocrinol Metab (2022) 33(9):601–13. doi: 10.1016/j.tem.2022.06.003 35872067

[5] Senesi P Luzi L Terruzzi I. Adipokines, myokines, and cardiokines: The role of nutritional interventions. Int J Mol Sci (2020) 21(21):8372. doi: 10.3390/ijms21218372 33171610 PMC7664629

[6] Kansra AR Lakkunarajah S Jay MS. Childhood and adolescent obesity: A review. Front Pediatr (2021) 8:581461. doi: 10.3389/fped.2020.581461 33511092 PMC7835259

